# Association between high temperature and mortality in metropolitan areas of four cities in various climatic zones in China: a time-series study

**DOI:** 10.1186/1476-069X-13-65

**Published:** 2014-08-07

**Authors:** Yonghong Li, Yibin Cheng, Guoquan Cui, Chaoqiong Peng, Yan Xu, Yulin Wang, Yingchun Liu, Jingyi Liu, Chengcheng Li, Zhen Wu, Peng Bi, Yinlong Jin

**Affiliations:** 1 Institute of Environmental Health and Related Product Safety, Chinese Center for Disease Control and Prevention, 29 Nanwei Road, Beijing 100021, China; 2 Harbin Center for Disease Control and Prevention, Harbin, China; 3 Shenzhen Center for Disease Control and Prevention, Shenzhen, China; 4 Jiangsu Provincial Center for Disease Control and Prevention, Nanjing, China; 5 Chongqing Center for Disease Control and Prevention, Chongqing, China; 6 Discipline of Public Health, School of Population Health and Clinical Practice, The University of Adelaide, Adelaide, Australia

**Keywords:** High temperature, Mortality, Vulnerable population, Risk assessment, China

## Abstract

**Background:**

Numerous studies have reported on the associations between ambient temperatures and mortality. However, few multi-city studies have been conducted in developing countries including China. This study aimed to examine the association between high temperature and mortality outcomes in four cities with different climatic characteristics in China to identify the most vulnerable population, detect the threshold temperatures, and provide scientific evidence for public health policy implementations to respond to challenges from extreme heat.

**Methods:**

A semi-parametric generalized additive model (GAM) with a Poisson distribution was used to analyze the impacts of the daily maximum temperature over the threshold on mortality after controlling for covariates including time trends, day of the week (DOW), humidity, daily temperature range, and outdoor air pollution.

**Results:**

The temperature thresholds for all-cause mortality were 29°C, 35°C, 33°C and 34°C for Harbin, Nanjing, Shenzhen and Chongqing, respectively. After adjusting for potential confounders including air pollution, strong associations between daily maximum temperature and daily mortality from all-cause, cardiovascular, endocrine and metabolic outcomes, and particularly diabetes, were observed in different geographical cities, with increases of 3.2-5.5%, 4.6-7.5% and 12.5-31.9% (with 14.7-29.2% in diabetes), respectively, with each 1°C increment in the daily maximum temperature over the threshold. A stronger temperature-associated mortality was detected in females compared to males. Additionally, both the population over 55 years and younger adults aged 30 to 54 years reported significant heat-mortality associations.

**Conclusions:**

Extreme heat is becoming a huge threat to public health and human welfare due to the strong temperature-mortality associations in China. Climate change with increasing temperatures may make the situation worse. Relevant public health strategies and an early extreme weather and health warning system should be developed and improved at an early stage to prevent and reduce the health risks due to extreme weather and climate change in China, given its huge population, diverse geographic distribution and unbalanced socioeconomic status with various climatic characteristics.

## Background

The average surface temperature of the earth has increased by approximately 0.8°C since 1900 and is projected to rise by 1.1 to 6.4°C during the next hundred years [1]. The average surface temperatures in China have also increased by 0.5–0.8°C over the past 100 years. According to the National Meteorological Bureau (NMB), China will continue to warm, with average temperatures increasing 1.3–2.1°C by the year 2020–2030 and 2.2-3.3°C by 2050. Increased temperatures can have direct and indirect impacts on human health.

Many previous studies have investigated the associations between heat and mortality in the United States [2-7], Europe [8-10], Australia [11-16], and Korea [17]. For example, a study in 15 European cities found that an increase of 3.12% (95% CI: 0.60-5.72%) in the Mediterranean region and 1.84% (95% CI: 0.06 to 3.64%) in the north-continental region in all natural mortality was associated with a 1°C increase in the maximum apparent temperature above the city-specific thresholds. Stronger associations were found between heat and mortality from respiratory diseases and deaths among the elderly [8]. Using the same method, studies conducted in California [5,6] and other parts of the USA [7] all reported that a 10°F increase in the apparent temperature was associated with approximately a 2% increase in mortality. The estimated effects of temperature on mortality may be heterogeneous across areas with differing climatic conditions, socioeconomic statuses and education levels. However, few studies have been conducted in developing countries including China, especially large scale multi-city studies involving different climatic zones.

Urban residents may be exposed to higher temperatures than residents of surrounding suburban and rural areas because of the “urban heat island effect”: this heat effect refers to the high thermal absorption by dark paved surfaces and buildings, heat emitted from vehicles and air conditioners, a lack of vegetation and trees, and poor ventilation [4,18]. As a result of the urban heat island effect, people in urban areas are usually at an increased risk of morbidity and mortality from ambient heat exposure [4]. The effects of temperature on mortality and morbidity are likely to become more severe because of the increasing elderly population among metropolitan areas in addition to the effects of climate change [19].

The purpose of this study was to estimate the heat thresholds for mortality outcomes in urban areas of four cities with different climate conditions in China, to examine the relationships between high temperatures and adverse health outcomes when adjusting for air quality to identify specific health outcomes that are sensitive to extreme heat and to provide scientific evidence for the establishment and implementation of public health policies to respond to health challenges from extreme heat and climate change.

## Methods

### Study area and climate

China has a range of temperature belts from the south to the north which are considered tropical, south subtropical, north subtropical, warm temperate, temperate, and frigid-temperate climate zones. The subtropical, warm temperate and temperate zones occupy 70% of the mainland.

**Figure 1:**
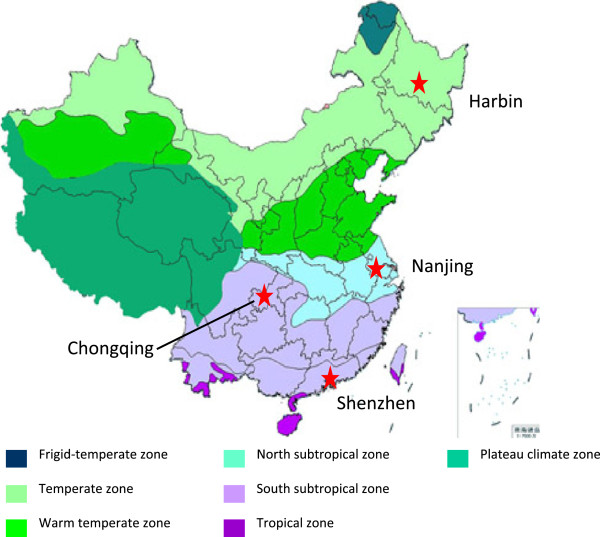
Geographical location of the 4 cities.

This study was conducted in the urban areas of four cities in China: Harbin, Nanjing, Shenzhen and Chongqing (Figure 1). Harbin (east longitude: 125°42′ to 130°10′, north latitude: 44°04′ to 46°40′) is located in the temperate zone: it has a continental monsoon climate, an average annual temperature of 5.6°C and an urban area of approximately 7,092 km^2^ with a population of 4.7 million in 2010 [20]. Harbin is the northernmost capital in China. Nanjing (east longitude: 118°22′ to 119°14′, north latitude: 31°14′ to 32°37′) belongs to a subtropical continental monsoon climate with an average annual temperature of 16.3°C and covers an urban area of 4,733 km^2^ with 3.1 million residents in 2010 [21]. Shenzhen (east longitude: 113°46′ to 114°37′, north latitude: 22°27′ to 22°52′) belongs to a subtropical marine monsoon climate with an average annual temperature of 23.0°C and has an urban area of 1,991 km^2^ with a population of 2.5 million in 2010 [22]. Chongqing (east longitude: 105°11′ to 110°11′, north latitude: 28°10′ to 32°13′) lies in south-western China, has a subtropical humid monsoon climate with an average annual temperature of 17.6°C, and covers an area of 82,400 km2 with approximately 15.2 million urban residents at the end of 2010 [23].

### Mortality data

The daily mortality data were collected from local Centers for Disease Control and Prevention, which is the official organization that collects and collates vital statistical data. The data covered different time periods for the four study cities because of data availability. The study periods were: Harbin (1 January 2008–31 December 2010), Nanjing (1 January 2004–31 December 2010), Shenzhen (1 January 2004–31 December 2010) and Chongqing (1 January 2011–31 December 2012). In the analyses, the following causes of death according to the *International Classification of Diseases 10* were used: all-cause (A00-Z99), cardiovascular (CVD, I00-I99), respiratory (J00-J99), digestive (K00-K93), endocrine and metabolic disease (E00-E90), and diabetes (E10-E14).

For Chongqing, on the basis of data availability, we used data from three districts (Yuzhong, Shapingba and Qijiang, with an estimated population of 1.8 million in 2011) for this study.

### Meteorological and air pollution data

The daily weather data were provided by the local Bureaus of Meteorology in each study city. The variables included daily maximum, mean and minimum temperatures (°C) in addition to the daily mean relative humidity (RH, %) for the same period as the mortality data.

The daily air quality data within the same period, including particulate matter of mass median aerodynamic diameter less than 10 μm (PM_10_, 24 hour mean in μg/m^3^), nitrogen dioxide (NO_2_, 24 hour mean in μg/m^3^) and sulfur dioxide (SO_2_, 24 hour mean in μg/m^3^), were obtained from the local Environmental Protection Bureaus of Harbin, Nanjing, Shenzhen and Chongqing.

### Statistical analysis

An observed/expected analysis was used for the estimation of the mortality heat thresholds, following the method of Dessai [24] and Williams [14]. A 31-day moving average, including 15 days before and after the index day, was used as an estimate of expected daily mortality. The observed daily values minus the expected values were calculated as the daily excess mortality. To associate excess mortality with daily temperature, the days were grouped into 1°C intervals of daily maximum temperature after rounding to the nearest whole degree, and the mean daily excess deaths were calculated for every temperature interval. The heat threshold was defined as the temperature interval below which excess events are not discernible [24].

A semi-parametric generalized additive model (GAM) with a Poisson distribution was used to analyze the associations between daily maximum temperatures over the thresholds and mortality outcomes, including all-cause, cardiovascular, respiratory, digestive, endocrine and metabolic mortalities, in addition to gender and age-specific mortalities. The temperature term in the GAM was the daily maximum temperature minus the threshold temperature: this term was set as 0 if the daily maximum temperature was lower than the threshold temperature. Only the warmer months, from 1 May to 30 September for Nanjing, Shenzhen and Chongqing and from 1 June through 31 August for Harbin, were considered in the analyses.

We used the daily maximum temperature in the analysis while most other studies have used the daily mean temperature. Because our study focused on the health impacts of high temperatures, the daily maximum temperature is the most direct indicator of a high temperature/heat wave. During heat waves, the minimum temperature at night is usually also a very important factor in deciding whether people can recover from the heat during the daytime, so we adjusted the daily temperature range, which is the daily maximum temperature minus the daily minimum temperature, in the model. Additionally, the weather forecast in China reports the daily maximum and minimum temperatures every day.

The relative humidity, daily range of temperature, day of the week (DOW), month, long-term trends, SO_2_, NO_2_, and PM_10_ were considered to be potential confounders. DOW and month were formatted as dummy variables in the model, and spline smoothed functions were applied to the other variables. The long-term trends were modeled with 6 degrees of freedom (df) per year. SO_2_, NO_2_, and PM_10_ were modeled with 4 degrees of freedom, and the daily temperature range was modeled with 2 df. The pairwise Pearson correlation coefficients for the different air pollutants ranged from 0.36 to 0.75: thus, multicollinearity was not a major concern [14]. The effect of temperature was estimated as the percent increase in daily mortality in relation to a 1°C increase in the daily maximum temperature. The relative risk (RR) and 95% confidence intervals (CI) were reported as the effect estimated.

### Sensitivity analysis

A sensitivity analysis was conducted by comparing the results with and without adjusting for the air pollutants and by using different degrees of freedom for time trends (5, 6 and 7 df) and air pollutants (3, 4 and 5 df) for each population group analyzed above (the results are shown in Additional file 1: Table S1–S3).

All the analyses were conducted using SAS 9.1 (SAS Institute Inc., Cary, NC, USA).

Ethical approval for this study was granted by the Institute of Environmental Health, Chinese Center for Disease Control and Prevention.

## Results

### Descriptive statistics

Table 1 shows the mean and range of daily deaths, temperature, relative humidity, and air pollutant concentrations for the four study areas. In these study areas and during the warm period, the mean number of daily deaths in Harbin, Nanjing, Shenzhen and Chongqing were 83.7 (min, max: 50, 126), 37.0 (min, max: 18, 71), 14.4 (min, max: 3, 31) and 34.9 (min, max: 16, 56), respectively. The ratio of male to female mean daily mortalities ranged from 1.2 to 1.8 in the four study areas. The deaths for individuals 65 years and older accounted for 61.4%, 75.4%, 40.3% and 69.1% of the total number of deaths in Harbin, Nanjing, Shenzhen and Chongqing, respectively. The mean daily maximum temperatures in the warm period studied were 27.4°C (min, max: 13.2, 37.1°C), 30.0°C (min, max: 16.5, 38.6°C), 31.4°C (min, max: 21.8, 37.9°C) and 29.9°C (min, max: 14.5, 40.9°C) in Harbin, Nanjing, Shenzhen and Chongqing, respectively.

**Table 1:** Descriptive statistics of daily mortality, meteorological parameters and air pollutants of the urban areas in 4 cities of China during the warm period

	**Harbin (2008-2010, June-Aug.)**				**Nanjing (2004-2010, May-Sep.)**				**Shenzhen (2004-2010, May-Sep.)**				**Chongqing (2011-2012, May-Sep.)**			
	**Mean ± SD**	**Max**	**Median**	**Min**	**Mean ± SD**	**Max**	**Median**	**Min**	**Mean ± SD**	**Max**	**Median**	**Min**	**Mean ± SD**	**Max**	**Median**	**Min**
All-cause mortality	83.7 ± 11.9	126	82.5	50	37.0 ± 7.1	71	37	18	14.4 ± 4.5	31	14	3	34.9 ± 7.8	56	35	16
Cardiovascular mortality (I00-I99)	37.9 ± 7.5	61	38	16	13.8 ± 4.1	31	14	2	3.4 ± 2.1	12	3	0	12.4 ± 4.0	26	12	4
Respiratory mortality (J00-J99)	9.1 ± 3.3	20	9	2	4.0 ± 1.9	12	4	1	0.8 ± 0.9	8	1	0	5.8 ± 2.6	14	5	1
Endocrine and metabolic mortality (E00-E90)	1.6 ± 1.1	6	2	1	1.7 ± 0.9	7	1	1	0.2 ± 0.5	4	0	0	1.7 ± 0.9	5	1	1
Diabetes mortality (E10-E14)	1.5 ± 1.1	6	1	0	1.6 ± 0.9	7	1	1	0.1 ± 0.2	2	0	0	1.6 ± 0.9	5	1	1
Digestive mortality (K00-K93)	2.7 ± 1.5	7	2	1	1.5 ± 0.8	6	1	1	0.4 ± 0.6	3	0	0	1.8 ± 1.0	6	1	1
Genitourinary mortality (N00-N99)	2.1 ± 1.1	6	2	1	1.3 ± 0.6	5	1	1	0.2 ± 0.4	3	0	0	1.2 ± 0.4	4	1	1
Male	50.1 ± 8.8	80	50	26	20.4 ± 5.0	38	20	5	9.3 ± 3.4	27	9	1	20.6 ± 5.4	36	21	6
Female	33.4 ± 6.5	54	33	18	16.6 ± 4.3	35	16	4	5.2 ± 2.4	14	5	1	14.2 ± 4.2	25	14	5
0-14 years	1.8 ± 1.0	7	2	1	1.3 ± 0.6	5	1	1	1.3 ± 0.6	5	1	1	1.4 ± 0.6	4	1	1
15-64 years	31.0 ± 6.3	54	31	14	8.7 ± 2.9	20	8	1	8.7 ± 3.5	22	8	1	10.5 ± 3.7	28	10	3
≥65 years	51.4 ± 8.6	82	51	31	27.9 ± 6.1	60	28	11	5.8 ± 2.4	15	5	2	24.1 ± 6.1	43	23	10
Maximum temp. (°C)	27.4 ± 3.5	37.1	27.6	13.2	30.0 ± 4.1	38.6	30.3	16.5	31.4 ± 2.4	37.9	31.8	21.8	29.9 ± 5.8	40.9	30.4	14.5
Mean temp. (°C)	22.5 ± 3.1	29.9	22.8	11.2	25.6 ± 3.7	34.5	25.8	14.3	28.1 ± 1.9	33	28.4	20.8	25.0 ± 4.6	34.9	24.9	13.3
Minimum temp. (°C)	17.8 ± 3.4	24.8	18	9	22.1 ± 3.9	30.8	22.6	9.4	25.7 ± 1.8	30.3	25.9	18.3	21.5 ± 3.8	30.6	21.4	12.3
Mean relative humidity (%)	72.1 ± 12.1	95	74	28	73.4 ± 11.6	98	74	37	76.1 ± 8.0	95	76	44	69.9 ± 16.4	94	71.8	26.1
Mean PM_10_ (μg/m^3^)	63.3 ± 37.4	175	57	7.5	95.1 ± 48.6	485.3	87.7	14	49.7 ± 26.1	186.4	41.4	13.1	73.2 ± 31.6	188.3	68.8	13.2
Mean SO_2_ (μg/m^3^)	22.5 ± 7.7	62	22.5	5	38.3 ± 17.8	114.7	35	5	19.7 ± 14.0	101.8	15.4	3.4	29.2 ± 11.6	70.5	28.6	8.7
Mean NO_2_ (μg/m^3^)	39.8 ± 23.7	148.5	31	11	43.2 ± 14.8	118.3	41	13.3	43.8 ± 19.3	138.2	38.8	13.1	31.4 ± 10.8	63.2	30.0	9.0
Area	Urban area of whole city				Urban area of whole city				Urban area of whole city				3 of 19 districts in urban area			

### Temperature thresholds for mortality

**Figure 2:**
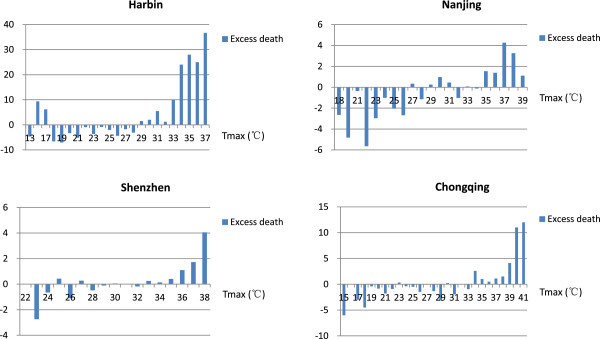
**Mean daily excess mortality associated with daily maximum temperatures in Harbin, Nanjing, Chongqing and Shenzhen, China.** The temperature thresholds, over which excess deaths significantly increase, were 29°C, 35°C, 33°C and 34°C for Harbin, Nanjing, Shenzhen and Chongqing, respectively.

Following the method constructed by Dessai [24] and Williams [13], the estimates for the heat thresholds for all-cause mortality were made using an observed/expected analysis because this accounts for time and seasonal trends in the data. Figure 2 shows the mean daily excess mortality for each 1°C interval of the daily maximum temperature of the four cities. The maximum temperature thresholds for the urban populations of Harbin, Nanjing, Shenzhen and Chongqing were estimated to be 29°C, 35°C, 33°C and 34°C, respectively, which are equivalent to 90%, 95%, 90% and 90% of the daily maximum temperature distributions of each specific city.

### Association between daily maximum temperature and mortalities

Table 2 summarizes the associations between the daily maximum temperature and the different mortality outcomes in all four study cities.

**Table 2:** Relationship of high temperature (Tmax) on mortality in four cities in China (the statistically significant results are bolded)

	**Harbin**			**Nanjing**			**Shenzhen**			**Chongqing**		
	**RR**	**95% CI**	**p**	**RR**	**95% CI**	**p**	**RR**	**95% CI**	**p**	**RR**	**95% CI**	**p**
All-cause	**1.045**	**(1.021,1.070)**	**0.001**	**1.032**	**(1.009,1.056)**	**0.007**	**1.040**	**(1.014,1.067)**	**0.003**	**1.055**	**(1.015,1.097)**	**0.010**
CVD	**1.046**	**(1.011,1.083)**	**0.012**	**1.050**	**(1.011,1.091)**	**0.012**	**1.075**	**(1.018,1.134)**	**0.009**	**1.069**	**(1.002,1.141)**	**0.049**
Respiratory	**1.080**	**(1.004,1.161)**	**0.048**	1.030	(0.955,1.110)	0.443	0.955	(0.837,1.089)	0.490	1.009	(0.914,1.115)	0.856
Digestive	1.117	(0.962,1.297)	0.158	1.106	(0.967,1.266)	0.149	1.088	(0.896,1.321)	0.396	**1.236**	**(1.029,1.485)**	**0.034**
Endocrine and metabolic	**1.232**	**(1.075,1.411)**	**0.016**	**1.125**	**(1.011,1.253)**	**0.034**	**1.319**	**(1.006,1.730)**	**0.046**	**1.236**	**(1.012,1.509)**	**0.050**
Diabetes	**1.252**	**(1.090,1.439)**	**0.002**	**1.147**	**(1.028,1.279)**	**0.015**	1.272	(0.695,2.328)	0.570	**1.292**	**(1.039,1.606)**	**0.026**
Male	**1.031**	**(1.002,1.062)**	**0.044**	**1.052**	**(1.020,1.085)**	**0.002**	**1.044**	**(1.011,1.078)**	**0.009**	**1.053**	**(1.004,1.104)**	**0.041**
Female	**1.072**	**(1.033,1.112)**	**0.001**	**1.057**	**(1.020,1.091)**	**0.002**	**1.046**	**(1.002,1.092)**	**0.039**	**1.062**	**(1.012,1.114)**	**0.021**
Age (years)												
0-14	1.047	(0.890,1.231)	0.585	1.005	(0.845,1.196)	0.955	1.015	(0.866,1.191)	0.851	0.934	(0.676,1.291)	0.687
0-5	1.092	(0.959,1.243)	0.193	1.037	(0.904,1.190)	0.606	**1.058**	**(1.017,1.100)**	**0.006**	0.964	(0.467,1.988)	0.926
15-29	1.008	(0.841,1.207)	0.935	1.016	(0.918,1.125)	0.759	1.050	(0.978,1.127)	0.176	1.014	(0.732,1.406)	0.933
30-54	**1.061**	**(1.004,1.121)**	**0.045**	0.965	(0.916,1.016)	0.174	**1.072**	**(1.015,1.131)**	**0.013**	**1.149**	**(1.045,1.263)**	**0.006**
55-64	**1.072**	**(1.024,1.122)**	**0.010**	**1.063**	**(1.013,1.116)**	**0.014**	1.035	(0.949,1.129)	0.434	**1.112**	**(1.003,1.233)**	**0.050**
65-74	**1.030**	**(1.002,1.060)**	**0.041**	**1.053**	**(1.013,1.095)**	**0.010**	1.008	(0.930,1.093)	0.848	**1.083**	**(1.004,1.168)**	**0.046**
≥75	**1.061**	**(1.022,1.101)**	**0.003**	**1.056**	**(1.020,1.071)**	**0.000**	**1.074**	**(1.003,1.149)**	**0.041**	**1.057**	**(1.009,1.107)**	**0.024**

Note: Male, female and age group specific results presented for all-cause mortality.

Significant associations between the daily maximum temperature and all-cause, cardiovascular, endocrine and metabolic mortalities were observed for all four cities. Above the threshold temperatures in each city, an increase of 1°C in the daily maximum temperature brought approximately a 4.5% (RR: 1.045, 95% CI: 1.021–1.070), 3.2% (RR: 1.032, 95% CI: 1.009–1.056), 4.0% (RR: 1.040, 95% CI: 1.014–1.067) and 5.5% (RR: 1.055, 95% CI: 1.015–1.097) increase in daily all-cause mortality in Harbin, Nanjing, Shenzhen and Chongqing, respectively. For cardiovascular diseases mortality, the increases in mortality were 4.6% (RR: 1.046, 95% CI: 1.011–1.083), 5.0% (RR: 1.050, 95% CI: 1.011–1.091), 7.5% (RR: 1.075, 95% CI: 1.018–1.134) and 6.9% (RR: 1.069, 95% CI: 1.002–1.141), respectively.

Furthermore, more significant associations were observed for increases in the daily endocrine and metabolic diseases mortality associated with a 1°C increase in the daily maximum temperature above the threshold, with mortality increase of 23.2% (RR: 1.232, 95% CI: 1.075–1.411), 12.5% (RR: 1.125, 95% CI: 1.011–1.253), 31.9% (RR: 1.319, 95% CI: 1.006–1.730) and 23.6% (RR: 1.236, 95% CI: 1.012–1.509) for Harbin, Nanjing, Shenzhen and Chongqing, respectively. The mortalities for diabetes and their associations with temperature were identified for further analysis, and an even larger increase was detected, with 25.2% (RR: 1.252, 95% CI: 1.090–1.439), 14.7% (RR: 1.147, 95% CI: 1.028–1.279) and 29.2% (RR 1.292, 95% CI: 1.039–1.606) increases in the daily diabetes mortality in Harbin, Nanjing and Chongqing, respectively. In addition, a 23.6% (RR: 1.236, 95% CI: 1.029–1.485) increase in daily digestive mortality in Chongqing and an 8.0% (RR: 1.080, 95% CI: 1.004–1.161) increase in respiratory mortality in Harbin were both significantly correlated with a 1°C increase in the daily maximum temperature over the threshold.

Both male and female deaths of all causes showed significant relationships with high temperatures and the relative risks (RR) for females were slightly higher than those for males in the four study areas.

The mortalities for the populations over 55 years showed significant associations with high temperatures in Harbin, Nanjing and Chongqing, and compared with the two groups of 65–74 years and those over 75 years, the group aged 55–64 years had a slightly higher increase in mortality corresponding to a 1°C increase in the daily maximum temperature above the threshold. In Harbin, Shenzhen and Chongqing, the mortalities for people of 30–54 years also increased by 6.1% (RR: 1.061, 95% CI: 1.004–1.121), 7.2% (RR: 1.072, 95% CI: 1.015–1.131) and 14.9% (RR: 1.149, 95% CI: 1.045–1.263), respectively, with a 1°C increase in the daily maximum temperature above the threshold, indicating that the working population is at risk from extreme heat as well. For the 0–5 year age group in Shenzhen, the RR of the temperature-mortality association was 1.058 (95% CI: 1.017–1.100), suggesting another group that is vulnerable to extreme heat.

## Discussion

We conducted this time-series study to assess the associations between high temperatures and urban mortality outcomes in four cities located in different climate zones of China, after controlling for covariates including time trends, day of the week (DOW), humidity, daily temperature range, and outdoor air pollution. To our best knowledge, this is the first such study in China that detects the threshold temperatures and assesses the impacts of extreme heat on human mortality. The results show significant associations between daily maximum temperatures and daily mortality after adjustment for potential confounders in all the study cities.

This multi-city study covers different climatic zones in China, which is one step further than the work by Wei et al. [25] that focused only on subtropical cities. More importantly, the effects of air pollution on human mortality have been controlled for in this study while most published studies have not done so. We believe it is important to control for the effect of air pollution because it contributes significantly to human mortality [26-29], especially in China, and we would suggest more such work in other cities of China, as well as in other Asian cities. We believe these factors are two innovations of this study.

Among the four study cities, the maximum temperature threshold estimate for mortality was the lowest at 29°C in the northernmost capital city of Harbin, 35°C and 34°C in two “oven” cities of Nanjing and Chongqing, respectively, which have hot and humid weather in the summer, and 33°C in Shenzhen. Roughly, compared with the northern city at higher latitude, the southern cities had higher temperature thresholds for the significant mortality increments. However, Shenzhen is the southernmost of the four cities, it is bordered by the South China Sea and the Pacific Ocean, which could regulate weather conditions against extremely high temperatures in summer. That might partly explain the moderate tolerance to higher temperatures in this population. The health-related temperature threshold is mainly related to the climate condition in each local region, public adaptation measures such as using air-conditioning and health education, knowledge of the health impacts of heat waves and self-protection, altitudes for learning or obtaining related knowledge among local residents, demographic characteristics, socioeconomic status, etc. Our results provide further support of geography-specific and climate-specific temperature thresholds for mortality, similar to other research studies [8,17,25,30-32], and suggest that city-specific early warning criteria against health risks from extreme heat should be developed, as opposed to a general criteria for all regions. The development and implementation of such a specific heat and health early warning system, based on the study results from each city, will obviously achieve greater health benefits.

Some studies have reported that high temperatures are related to an increased risk of non-accidental and cardiovascular mortality [3,7,25,32-35]. Our results are consistent with previous findings and reported significant associations between high temperatures and all-cause and cardiovascular mortalities in all four of the study cities with different climatic conditions, with stronger associations for cardiovascular mortalities than for all-cause mortalities. Exposure to extremely hot temperatures might induce dehydration, cause salt depletion and increase surface blood circulation, which can lead to a failure of thermoregulation [36]. Extremely hot temperatures may also be related to elevated blood viscosity, cholesterol levels and sweating thresholds [37]. Our results contribute to evidence demonstrating that extreme heat can increase mortality, and people suffering from diseases, particularly cardiovascular diseases, seem to be more vulnerable to high temperature effects.

It has been previously reported that hot days or increases in temperature in the summer were associated with increases in endocrine and metabolic morbidity, particularly for diabetes [2,12,15,38]. Our study reported, for the first time, that there are significant associations between ambient temperature and endocrine and metabolic mortality and diabetes mortality (*which accounted for 95.5%, 94.3%, 20.0% and 90.2% of the endocrine and metabolic mortality during the warm period in Harbin, Nanjing, Shenzhen and Chongqing, respectively*) in Chinese populations among different climate-specific cities. Some previous studies found that ambient temperature apparently affected glucose tolerance [39,40], which was most likely due to the redistribution of blood flow between cutaneous and visceral beds driven by changes in core temperature because of the ambient temperature changes [41-43]. Additionally, Koivisto’s findings indicated that a rise in ambient temperature could augment insulin absorption in insulin-treated diabetic patients [39]. A potential explanation for our findings is that diabetic patients may die from the adverse outcomes of being unable to maintain a stable plasma glucose level due to more blood flow to cutaneous areas and less blood flow to visceral beds, augmented with insulin absorption caused by changes in core temperature on extremely hot days. Therefore, relevant health intervention measurements should be implemented that specifically target such patients, especially during hot days. This obviously needs a joint initiative between clinicians, health promotion staff and the local community. The non-significant results for temperature-diabetes mortality in Shenzhen may be due to the small number of daily mortalities in that city.

It is very significant that considerable associations between temperature and all-cause, cardiovascular, endocrine and metabolic mortality were observed in all four study cities. Significant associations for respiratory and digestive mortality were also observed in some cities. These chronic diseases mentioned above are the most common causes of morbidity in China, especially for people over 65 years old, with prevalence rates of 153.3‰ for cardiovascular, 31.4‰ for endocrine and metabolic (diabetes accounted for 27.5‰), 21.9‰ for digestive and 15.7‰ for respiratory diseases in the urban population in 2008 [44], which indicates that there is a large population at risk of dying from high temperatures. Our results found that increases in mortality from these chronic diseases were significantly associated with extremely high temperatures in geography- and climate –specific cities. This indicates that extreme heat is becoming a huge threat for public health and human welfare, especially with the increasing elderly society in China.

Many previous studies have reported a higher susceptibility to high temperature/heat waves among the elderly [1,3,8,17,45], with the explanation that aging results in physiological changes in thermoregulation and homeostasis, together with the increased prevalence of chronic diseases and the use of medication, thus inducing a susceptibility to heat stress [1]. However, Nitschke et al. found that the 2009 heat wave in Adelaide, Australia, was associated with considerable increases in total mortality that particularly affected the 15–64 year age group (1.37; 95% CI: 1.09, 1.71), while older age groups were unaffected [16]. A study on the impact of heat on mortality in 15 European cities suggested that the largest impact was on persons over 75 years; however, in some cities, important proportions of heat-attributable deaths were also reported for younger adults [9]. The results of our study conducted in four different geographical and climatic cities displayed that, in addition to the group older than 55 years, people aged 30–54 years also had apparently significant associations in total mortality with extremely high temperatures. This age range may have more chances to be exposed to extreme heat, particularly when they conduct outdoor work during extremely hot days, thus creating the association with mortality even though they may have relatively better capacities of thermoregulation and heat tolerance than the elderly. Indeed, the sudden death of outdoor workers during the summer of 2012 in several cities of China suggested that relevant regulations and practical guidelines should be developed to protect these “healthy workers”. The inconsistency in the age group of the vulnerable population may be partly due to the different age grouping methods of various studies. These findings suggest that both exposure and the capacity of thermoregulation and heat tolerance are key risk factors contributing to the adverse health outcomes associated with extremely high temperatures. This may have implications for developing and improving adaptation strategies against extreme heat.

We also found significant heat-related increases in mortality for children younger than 5 years in Shenzhen. While most of the previous studies focused on adults and very few have considered children [6,46], investigators have reported that the health effects of children must be considered in climate change research [47]. Children are often the most vulnerable to adverse health effects from environmental hazards, including extreme heat, because of their physical, physiologic, and cognitive immaturity [47]. More large-sample studies are necessary in this field, and our results suggest that further research to associate heat with health effects in children is warranted.

We observed significantly (*in Harbin*) or slightly (*in Nanjing, Shenzhen and Chongqing*) stronger temperature-associated mortalities in females than in males. This gender difference was also observed in previous epidemiological studies [1,3,48], and experimental evidence also demonstrates that females are more heat intolerant than males because of potential gender-related physiological and thermoregulatory differences [49,50].

It should be noted that mortality was the only health outcome used in this study. In fact, morbidity indexes such as hospitalizations, ambulance call-outs and emergency department visits are more sensitive to heat and should be used to assess the association between extreme heat and population health for the next step depending on the availability of these data. Another limitation of this study is that different assessment periods were used for the four cities due to limited data availability, which induces implications for the comparability of the results.

## Conclusions

Our results indicate that there were significant increases in all-cause mortality, especially for cardiovascular, endocrine and metabolic diseases, with extreme heat in temperate and subtropical cities of China. Further examination of adverse health outcomes, the identification of vulnerable populations and strategies to protect them from extreme heat could have immediate and future health benefits for the urban population of China. The adverse health impacts of hot weather are largely preventable, and the results have profound implications for developing early warning systems and response planning against the more frequent and extreme hot weather projected for the urban population of China.

## Abbreviations

GAM: Generalized additive model; DOW: Day of the week; NMB: National Meteorological Bureau; CVD: Cardiovascular diseases; RH: Relative humidity; PM_10_: Particulate matter of mass median aerodynamic diameter less than 10 μm; NO_2_: Nitrogen dioxide; SO_2_: Sulfur dioxide; RR: Relative risk; CI: Confidence interval.

## Competing interests

The authors declare that they have no competing interests.

## Authors’ contributions

YHL contributed to writing the manuscript, the study concept and design, and the analysis and interpretation of the data. YBC, GQC, CQP, YX and YLW contributed to writing the manuscript and the analysis and interpretation of the data. YCL, JYL, and ZW contributed to writing the manuscript. PB contributed to writing the manuscript, the study concept and design, and the interpretation of the data. YLJ contributed to writing the manuscript, the study concept and design, and the analysis and interpretation of the data. All authors read and approved the final manuscript.

## Supplementary Material

Additional file 1 **Sensitivity analysis results. Table S1.** Relationship of high temperature (Tmax) on mortality with and without adjusting of air pollutants of four cities in China (the statistically significant results are bolded). **Table S2.** Relationship of high temperature (Tmax) on mortality with different df for long-term trend (date) of four cities in China (the statistically significant results are bolded). **Table S3.** Relationship of high temperature (Tmax) on mortality with different df for air pollutants of four cities in China (the statistically significant results are bolded). Click here for file
