# Supplementary material for: Association between high temperature and mortality in metropolitan areas of four cities in various climatic zones in China: a time-series study
Source: Environ Health. 2014 Aug 7;13:65. doi: 10.1186/1476-069X-13-65 (PMC4237799; doi:10.1186/1476-069X-13-65)
Supplement: Additional file 1 — Sensitivity analysis results. Table S1. Relationship of high temperature (Tmax) on mortality with and without adjusting of air pollutants of four cities in China (the statistically significant results are bolded). Table S2. Relationship of high temperature (Tmax) on mortality with different df for long-term trend (date) of four cities in China (the statistically significant results are bolded). Table S3. Relationship of high temperature (Tmax) on mortality with different df for air pollutants of four cities in China (the statistically significant results are bolded). [file 1476-069X-13-65-S1.zip › 1688859871246038_TableS1.pdf]

Table S1 Relationship of high temperature (Tmax) on mortality with and without adjusting of air pollutants of four cities in China (the statistically significant results are bolded)

	Harbin						Nanjing						Shenzhen						Chongqing					
	Adjusted air pollution			Un-adjusted air pollution			Adjusted air pollution			Un-adjusted air pollution			Adjusted air pollution			Un-adjusted air pollution			Adjusted air pollution			Un-adjusted air pollution		
	RR	95% CI	p	RR	95% CI	p	RR	95% CI	p	RR	95% CI	p	RR	95% CI	p	RR	95% CI	p	RR	95% CI	p	RR	95% CI	p
All-cause	**1.045**	**(1.021,1.070)**	**0.001**	**1.044**	**(1.023,1.065)**	**0.000**	**1.032**	**(1.009,1.056)**	**0.007**	**1.031**	**(1.010,1.053)**	**0.004**	**1.040**	**(1.014,1.067)**	**0.003**	**1.034**	**(1.003,1.067)**	**0.033**	**1.055**	**(1.015,1.097)**	**0.010**	**1.068**	**(1.030,1.109)**	**0.001**
CVD	**1.046**	**(1.011,1.083)**	**0.012**	**1.039**	**(1.009,1.070)**	**0.014**	**1.050**	**(1.011,1.091)**	**0.012**	**1.045**	**(1.010,1.082)**	**0.012**	**1.075**	**(1.018,1.134)**	**0.009**	**1.062**	**(1.010,1.115)**	**0.018**	**1.069**	**(1.002,1.141)**	**0.050**	**1.070**	**(1.006,1.138)**	**0.037**
Respiratory	**1.080**	**(1.004,1.161)**	**0.048**	**1.077**	**(1.013,1.146)**	**0.021**	**1.030**	**(0.955,1.110)**	**0.443**	**1.024**	**(0.957,1.095)**	**0.497**	**0.955**	**(0.837,1.089)**	**0.490**	**0.991**	**(0.877,1.120)**	**0.886**	**1.009**	**(0.914,1.115)**	**0.856**	**1.002**	**(0.912,1.102)**	**0.964**
Digestive	1.117	(0.962,1.297)	0.158	1.057	(0.934,1.196)	0.382	1.106	(0.967,1.266)	0.149	1.072	(0.947,1.214)	0.273	1.088	(0.896,1.321)	0.396	1.074	(0.908,1.271)	0.404	1.236	(1.029,1.485)	0.035	1.212	(1.008,1.458)	0.049
Endocrine and metabolic	**1.232**	**(1.075,1.411)**	**0.016**	**1.222**	**(1.079,1.383)**	**0.003**	**1.125**	**(1.011,1.253)**	**0.034**	**1.064**	**(0.960,1.179)**	**0.239**	**1.319**	**(1.006,1.730)**	**0.046**	**1.057**	**(0.835,1.338)**	**0.645**	**1.236**	**(1.012,1.509)**	**0.050**	**1.230**	**(1.011,1.497)**	**0.055**
Diabetes	**1.252**	**(1.090,1.439)**	**0.002**	**1.169**	**(1.038,1.317)**	**0.012**	**1.147**	**(1.028,1.279)**	**0.015**	**1.070**	**(0.963,1.189)**	**0.212**	**1.272**	**(0.695,2.328)**	**0.571**	**1.135**	**(0.507,2.545)**	**0.771**	**1.292**	**(1.039,1.606)**	**0.026**	**1.333**	**(1.061,1.675)**	**0.018**
Male	**1.031**	**(1.002,1.062)**	**0.044**	**1.028**	**(1.002,1.055)**	**0.038**	**1.052**	**(1.020,1.085)**	**0.002**	**1.028**	**(0.999,1.057)**	**0.061**	**1.044**	**(1.011,1.078)**	**0.009**	**1.035**	**(1.006,1.066)**	**0.020**	**1.053**	**(1.004,1.104)**	**0.041**	**1.067**	**(1.017,1.119)**	**0.012**
Female	**1.072**	**(1.033,1.112)**	**0.001**	**1.069**	**(1.035,1.104)**	**0.000**	**1.057**	**(1.022,1.094)**	**0.002**	**1.059**	**(1.027,1.092)**	**0.000**	**1.046**	**(1.002,1.092)**	**0.039**	**1.042**	**(1.002,1.083)**	**0.039**	**1.062**	**(1.012,1.114)**	**0.021**	**1.052**	**(1.003,1.103)**	**0.042**
Age (years)																								
0-14	1.047	(0.890,1.231)	0.585	1.097	(0.956,1.260)	0.194	1.005	(0.845,1.196)	0.955	0.983	(0.833,1.161)	0.845	1.015	(0.866,1.191)	0.851	1.029	(0.893,1.187)	0.691	0.934	(0.676,1.291)	0.687	0.936	(0.601,1.457)	0.800
0-5	1.092	(0.959,1.243)	0.193	1.120	(0.992,1.264)	0.075	1.037	(0.904,1.19)	0.606	1.033	(0.911,1.171)	0.616	1.071	(0.852,1.347)	0.556	1.067	(0.868,1.312)	0.538	0.964	(0.467,1.988)	0.927	1.057	(0.506,2.210)	0.886
15-29	1.008	(0.841,1.207)	0.935	1.029	(0.876,1.210)	0.728	1.016	(0.918,1.125)	0.759	1.007	(0.913,1.111)	0.888	1.050	(0.978,1.127)	0.176	1.046	(0.982,1.115)	0.161	1.014	(0.732,1.406)	0.933	1.015	(0.746,1.383)	0.924
30-54	**1.061**	**(1.004,1.121)**	**0.045**	**1.079**	**(1.029,1.132)**	**0.004**	**0.965**	**(0.916,1.016)**	**0.174**	**0.957**	**(0.912,1.003)**	**0.070**	**1.072**	**(1.015,1.131)**	**0.013**	**1.038**	**(0.988,1.091)**	**0.142**	**1.149**	**(1.045,1.263)**	**0.006**	**1.149**	**(1.047,1.261)**	**0.005**
55-64	**1.072**	**(1.024,1.122)**	**0.010**	1.021	(0.981,1.063)	0.309	**1.063**	**(1.013,1.116)**	**0.014**	1.052	(1.007,1.100)	0.024	**1.035**	**(0.949,1.129)**	**0.434**	1.017	(0.943,1.098)	0.656	**1.112**	**(1.003,1.233)**	**0.050**	1.112	(1.006,1.228)	0.044
65-74	**1.030**	**(1.002,1.060)**	**0.041**	**1.029**	**(1.003,1.055)**	**0.030**	**1.053**	**(1.013,1.095)**	**0.010**	**1.046**	**(1.010,1.084)**	**0.013**	**1.008**	**(0.930,1.093)**	**0.848**	**1.007**	**(0.937,1.083)**	**0.848**	**1.083**	**(1.004,1.168)**	**0.046**	**1.109**	**(1.031,1.193)**	**0.008**
≥75	**1.061**	**(1.022,1.101)**	**0.003**	**1.064**	**(1.030,1.099)**	**0.001**	**1.056**	**(1.024,1.089)**	**0.001**	**1.055**	**(1.026,1.084)**	**0.000**	**1.074**	**(1.003,1.149)**	**0.041**	**1.065**	**(1.002,1.132)**	**0.044**	**1.057**	**(1.009,1.107)**	**0.024**	**1.065**	**(1.015,1.118)**	**0.014**

Note: Male, female and age group specific results presented for all-cause mortality.
