# Supplementary material for: Association between high temperature and mortality in metropolitan areas of four cities in various climatic zones in China: a time-series study
Source: Environ Health. 2014 Aug 7;13:65. doi: 10.1186/1476-069X-13-65 (PMC4237799; doi:10.1186/1476-069X-13-65)
Supplement: Additional file 1 — Sensitivity analysis results. Table S1. Relationship of high temperature (Tmax) on mortality with and without adjusting of air pollutants of four cities in China (the statistically significant results are bolded). Table S2. Relationship of high temperature (Tmax) on mortality with different df for long-term trend (date) of four cities in China (the statistically significant results are bolded). Table S3. Relationship of high temperature (Tmax) on mortality with different df for air pollutants of four cities in China (the statistically significant results are bolded). [file 1476-069X-13-65-S1.zip › 1688859871246038_TableS2.pdf]

Table S2 Relationship of high temperature (Tmax) on mortality with different df for long-term trend (date) of four cities in China (the statistically significant results are bolded)

	Harbin									Nanjing								
	date df=5/year			date df=6/year			date df=7/year			date,df=5			date,df=6			date,df=7		
	RR	95% CI	p	RR	95% CI	p	RR	95% CI	p	RR	95% CI	p	RR	95% CI	p	RR	95% CI	p
All-cause	**1.046**	**(1.022,1.071)**	**0.001**	**1.045**	**(1.021,1.070)**	**0.001**	**1.043**	**(1.019,1.068)**	**0.001**	**1.030**	**(1.006,1.053)**	**0.014**	**1.032**	**(1.009,1.056)**	**0.007**	**1.034**	**(1.01,1.057)**	**0.005**
CVD	**1.047**	**(1.012,1.083)**	**0.011**	**1.046**	**(1.011,1.083)**	**0.012**	**1.046**	**(1.011,1.082)**	**0.014**	**1.053**	**(1.013,1.093)**	**0.009**	**1.050**	**(1.011,1.091)**	**0.012**	**1.053**	**(1.014,1.094)**	**0.008**
Respiratory	**1.083**	**(1.007,1.164)**	**0.041**	**1.080**	**(1.004,1.161)**	**0.048**	**1.077**	**(1.001,1.158)**	**0.055**	**1.023**	**(0.950,1.102)**	**0.543**	**1.030**	**(0.955,1.110)**	**0.443**	**1.030**	**(0.956,1.109)**	**0.438**
Digestive	1.046	(0.902,1.212)	0.558	1.117	(0.962,1.297)	0.158	1.047	(0.904,1.213)	0.545	1.100	(0.961,1.258)	0.173	1.106	(0.967,1.266)	0.149	1.106	(0.966,1.267)	0.151
Endocrine and metabolic	**1.234**	**(1.077,1.414)**	**0.012**	**1.232**	**(1.075,1.411)**	**0.016**	**1.227**	**(1.071,1.406)**	**0.023**	**1.103**	**(0.991,1.228)**	**0.075**	**1.125**	**(1.011,1.253)**	**0.034**	**1.133**	**(1.018,1.262)**	**0.025**
Diabetes	**1.259**	**(1.096,1.447)**	**0.002**	**1.252**	**(1.090,1.439)**	**0.002**	**1.251**	**(1.090,1.437)**	**0.002**	**1.123**	**(1.006,1.254)**	**0.040**	**1.147**	**(1.028,1.279)**	**0.015**	**1.163**	**(1.042,1.297)**	**0.008**
Male	**1.032**	**(1.003,1.063)**	**0.038**	**1.031**	**(1.002,1.062)**	**0.044**	**1.029**	**(1.000,1.060)**	**0.057**	**1.047**	**(1.015,1.080)**	**0.004**	**1.052**	**(1.020,1.085)**	**0.002**	**1.056**	**(1.024,1.089)**	**0.001**
Female	**1.073**	**(1.034,1.114)**	**0.001**	**1.072**	**(1.033,1.112)**	**0.001**	**1.067**	**(1.028,1.107)**	**0.001**	**1.051**	**(1.016,1.087)**	**0.004**	**1.055**	**(1.020,1.091)**	**0.002**	**1.057**	**(1.022,1.093)**	**0.002**
Age (years)																		
0-14	1.049	(0.887,1.240)	0.583	1.047	(0.890,1.231)	0.585	1.043	(0.882,1.233)	0.629	0.937	(0.788,1.115)	0.473	1.005	(0.845,1.196)	0.955	0.915	(0.768,1.089)	0.327
0-5	1.068	(0.934,1.221)	0.343	1.092	(0.959,1.243)	0.193	1.079	(0.943,1.234)	0.274	1.045	(0.911,1.199)	0.540	1.037	(0.904,1.190)	0.606	1.029	(0.897,1.180)	0.688
15-29	1.003	(0.838,1.201)	0.972	1.008	(0.841,1.207)	0.935	1.010	(0.843,1.210)	0.914	1.013	(0.915,1.122)	0.805	1.016	(0.918,1.125)	0.759	1.016	(0.887,1.165)	0.828
30-54	1.048	(0.992,1.108)	0.099	**1.061**	**(1.004,1.121)**	**0.045**	**1.059**	**(1.003,1.120)**	**0.051**	0.964	(0.916,1.015)	0.164	**0.965**	**(0.916,1.016)**	**0.174**	**0.966**	**(0.918,1.017)**	**0.192**
55-64	1.090	(1.041,1.141)	0.253	**1.072**	**(1.024,1.122)**	**0.010**	**1.070**	**(1.022,1.120)**	**0.011**	1.061	(1.010,1.114)	0.018	**1.063**	**(1.013,1.116)**	**0.014**	**1.074**	**(1.023,1.127)**	**0.005**
65-74	**1.030**	**(1.001,1.06)**	**0.041**	**1.030**	**(1.002,1.060)**	**0.041**	**1.030**	**(1.002,1.060)**	**0.040**	**1.051**	**(1.011,1.093)**	**0.013**	**1.053**	**(1.013,1.095)**	**0.010**	**1.058**	**(1.018,1.100)**	**0.005**
≥75	**1.058**	**(1.019,1.097)**	**0.005**	**1.061**	**(1.022,1.101)**	**0.003**	**1.063**	**(1.025,1.103)**	**0.002**	**1.057**	**(1.025,1.090)**	**0.001**	**1.056**	**(1.024,1.089)**	**0.001**	**1.052**	**(1.020,1.085)**	**0.002**

	Shenzhen									Chongqing								
	date,df=5			date,df=6			date,df=7			date，df=5			date，df=6			date，df=7		
	RR	95% CI	p	RR	95% CI	p	RR	95% CI	p	RR	95% CI	p	RR	95% CI	p	RR	95% CI	p
All-cause	**1.041**	**(1.006,1.077)**	**0.023**	**1.040**	**(1.014,1.067)**	**0.003**	**1.039**	**(1.004,1.075)**	**0.031**	**1.057**	**(1.016,1.098)**	**0.008**	**1.055**	**(1.015,1.097)**	**0.010**	**1.056**	**(1.016,1.098)**	**0.009**
CVD	**1.067**	**(1.01,1.126)**	**0.020**	**1.075**	**(1.018,1.134)**	**0.009**	**1.063**	**(1.007,1.122)**	**0.028**	**1.037**	**(0.971,1.106)**	**0.285**	**1.069**	**(1.002,1.141)**	**0.050**	**1.077**	**(1.009,1.149)**	**0.031**
Respiratory	**0.943**	**(0.826,1.075)**	**0.380**	**0.955**	**(0.837,1.089)**	**0.490**	**0.936**	**(0.821,1.067)**	**0.323**	**1.009**	**(0.913,1.115)**	**0.859**	**1.009**	**(0.914,1.115)**	**0.856**	**1.006**	**(0.910,1.111)**	**0.909**
Digestive	1.076	(0.887,1.305)	0.460	1.088	(0.896,1.321)	0.396	1.055	(0.868,1.281)	0.592	1.225	(1.020,1.472)	0.042	1.236	(1.029,1.485)	0.035	1.213	(1.010,1.457)	0.051
Endocrine and metabolic	**1.201**	**(0.921,1.567)**	**0.176**	**1.319**	**(1.006,1.73)**	**0.046**	**1.332**	**(1.015,1.747)**	**0.039**	**1.230**	**(1.008,1.502)**	**0.054**	**1.236**	**(1.012,1.509)**	**0.050**	**1.234**	**(1.011,1.507)**	**0.053**
Diabetes	**1.362**	**(0.568,3.265)**	**0.530**	**1.272**	**(0.695,2.328)**	**0.571**	**1.135**	**(0.611,2.108)**	**0.713**	**1.323**	**(1.064,1.647)**	**0.016**	**1.292**	**(1.039,1.606)**	**0.026**	**1.306**	**(1.050,1.624)**	**0.021**
Male	**1.041**	**(1.009,1.075)**	**0.013**	**1.044**	**(1.011,1.078)**	**0.009**	**1.039**	**(1.007,1.073)**	**0.018**	**1.050**	**(1.001,1.101)**	**0.052**	**1.053**	**(1.004,1.104)**	**0.041**	**1.060**	**(1.011,1.112)**	**0.021**
Female	**1.048**	**(1.004,1.094)**	**0.031**	**1.046**	**(1.002,1.092)**	**0.039**	**1.033**	**(0.99,1.078)**	**0.062**	**1.057**	**(1.007,1.109)**	**0.032**	**1.062**	**(1.012,1.114)**	**0.021**	**1.052**	**(1.002,1.104)**	**0.048**
Age (years)																		
0-14	1.011	(0.862,1.185)	0.894	1.015	(0.866,1.191)	0.851	1.007	(0.860,1.181)	0.927	0.917	(0.665,1.265)	0.606	0.934	(0.676,1.291)	0.687	0.949	(0.686,1.312)	0.757
0-5	1.080	(0.859,1.357)	0.514	1.071	(0.852,1.347)	0.556	1.077	(0.857,1.353)	0.530	0.932	(0.451,1.925)	0.866	0.964	(0.467,1.988)	0.927	0.979	(0.477,2.008)	0.956
15-29	1.057	(0.985,1.134)	0.128	1.050	(0.978,1.127)	0.176	1.056	(0.984,1.133)	0.133	1.017	(0.733,1.410)	0.921	1.014	(0.732,1.406)	0.933	1.016	(0.733,1.409)	0.924
30-54	1.075	(1.018,1.134)	0.010	**1.072**	**(1.015,1.131)**	**0.013**	**1.075**	**(1.018,1.134)**	**0.009**	1.149	(1.045,1.262)	0.006	**1.149**	**(1.045,1.263)**	**0.006**	**1.151**	**(1.046,1.266)**	**0.006**
55-64	1.022	(0.937,1.115)	0.628	**1.035**	**(0.949,1.129)**	**0.434**	**1.020**	**(0.935,1.113)**	**0.654**	1.115	(1.006,1.236)	0.044	**1.112**	**(1.003,1.233)**	**0.050**	**1.108**	**(0.999,1.228)**	**0.059**
65-74	**1.004**	**(0.926,1.089)**	**0.918**	**1.008**	**(0.930,1.093)**	**0.848**	**1.006**	**(0.928,1.091)**	**0.876**	**1.071**	**(0.993,1.155)**	**0.084**	**1.083**	**(1.004,1.168)**	**0.046**	**1.089**	**(1.010,1.174)**	**0.033**
≥75	**1.072**	**(1.001,1.147)**	**0.047**	**1.074**	**(1.003,1.149)**	**0.041**	**1.076**	**(1.005,1.152)**	**0.036**	**1.052**	**(1.004,1.102)**	**0.037**	**1.057**	**(1.009,1.107)**	**0.024**	**1.047**	**(0.999,1.097)**	**0.058**

Note: Male, female and age group specific results presented for all-cause mortality.
