# Supplementary material for: Association between high temperature and mortality in metropolitan areas of four cities in various climatic zones in China: a time-series study
Source: Environ Health. 2014 Aug 7;13:65. doi: 10.1186/1476-069X-13-65 (PMC4237799; doi:10.1186/1476-069X-13-65)
Supplement: Additional file 1 — Sensitivity analysis results. Table S1. Relationship of high temperature (Tmax) on mortality with and without adjusting of air pollutants of four cities in China (the statistically significant results are bolded). Table S2. Relationship of high temperature (Tmax) on mortality with different df for long-term trend (date) of four cities in China (the statistically significant results are bolded). Table S3. Relationship of high temperature (Tmax) on mortality with different df for air pollutants of four cities in China (the statistically significant results are bolded). [file 1476-069X-13-65-S1.zip › 1688859871246038_TableS3.pdf]

Table S3 Relationship of high temperature (Tmax) on mortality with different df for air pollutants of four cities in China (the statistically significant results are bolded)

Harbin	air pollutants df=3/year			air pollutants df=4/year			air pollutants df=5/year		
	RR	95% CI	p	RR	95% CI	p	RR	95% CI	p
All-cause	**1.043**	**(1.019,1.068)**	**0.002**	**1.045**	**(1.021,1.070)**	**0.001**	**1.046**	**(1.021,1.071)**	**0.004**
CVD	**1.054**	**(1.019,1.091)**	**0.004**	**1.046**	**(1.011,1.083)**	**0.012**	**1.064**	**(1.028,1.101)**	**0.001**
Respiratory	**1.093**	**(1.017,1.175)**	**0.021**	**1.080**	**(1.004,1.161)**	**0.048**	**1.108**	**(1.031,1.192)**	**0.010**
Digestive	1.078	(0.932,1.248)	0.321	1.117	(0.962,1.297)	0.158	1.093	(0.944,1.265)	0.241
Endocrine and metabolic	**1.232**	**(1.074,1.413)**	**0.007**	**1.232**	**(1.075,1.411)**	**0.016**	**1.239**	**(1.080,1.420)**	**0.007**
Diabetes	**1.369**	**(1.195,1.568)**	**0.000**	**1.252**	**(1.090,1.439)**	**0.002**	**1.252**	**(1.090,1.437)**	**0.003**
Male	**1.031**	**(1.001,1.061)**	**0.054**	**1.031**	**(1.002,1.062)**	**0.044**	**1.036**	**(1.006,1.067)**	**0.030**
Female	**1.065**	**(1.026,1.106)**	**0.005**	**1.072**	**(1.033,1.112)**	**0.001**	**1.065**	**(1.026,1.106)**	**0.033**
Age (years)									
0-14	1.006	(0.850,1.190)	0.949	1.047	(0.887,1.240)	0.585	1.021	(0.863,1.209)	0.810
0-5	1.017	(0.889,1.162)	0.813	1.092	(0.959,1.243)	0.193	1.031	(0.902,1.179)	0.657
15-29	1.028	(0.999,1.057)	0.058	1.008	(0.841,1.207)	0.935	1.016	(0.878,1.177)	0.830
30-54	**1.065**	**(1.009,1.125)**	**0.033**	**1.061**	**(1.004,1.121)**	**0.045**	**1.064**	**(1.007,1.124)**	**0.033**
55-64	**1.050**	**(1.003,1.099)**	**0.042**	**1.072**	**(1.024,1.122)**	**0.010**	**1.046**	**(1.000,1.095)**	**0.060**
65-74	1.025	(0.996,1.054)	0.090	**1.030**	**(1.002,1.060)**	**0.041**	**1.023**	(0.994,1.052)	0.123
≥75	**1.066**	**(1.027,1.107)**	**0.003**	**1.061**	**(1.022,1.101)**	**0.003**	**1.080**	**(1.040,1.121)**	**0.001**

Nanjing	air pollution, df=3			air pollution, df=4			air pollution, df=5		
	RR	95% CI	p	RR	95% CI	p	RR	95% CI	p
All-cause	**1.031**	**(1.007,1.055)**	**0.010**	**1.032**	**(1.009,1.056)**	**0.007**	**1.029**	**(1.006,1.053)**	**0.015**
CVD	**1.052**	**(1.013,1.093)**	**0.010**	**1.050**	**(1.011,1.091)**	**0.012**	**1.048**	**(1.009,1.089)**	**0.017**
Respiratory	**1.029**	**(0.954,1.109)**	**0.461**	**1.030**	**(0.955,1.110)**	**0.443**	**1.027**	**(0.953,1.107)**	**0.490**
Digestive	1.066	(0.932,1.220)	0.353	1.106	(0.967,1.266)	0.149	1.065	(0.930,1.218)	0.365
Endocrine and metabolic	**1.121**	**(1.006,1.248)**	**0.041**	**1.125**	**(1.011,1.253)**	**0.034**	**1.122**	**(1.007,1.249)**	**0.040**
Diabetes	**1.145**	**(1.026,1.278)**	**0.017**	**1.147**	**(1.028,1.279)**	**0.015**	**1.142**	**(1.023,1.274)**	**0.019**
Male	**1.047**	**(1.015,1.080)**	**0.004**	**1.052**	**(1.020,1.085)**	**0.002**	**1.043**	**(1.011,1.076)**	**0.009**
Female	**1.048**	**(1.013,1.084)**	**0.007**	**1.055**	**(1.020,1.091)**	**0.002**	**1.047**	**(1.013,1.083)**	**0.008**
Age (years)									
0-14	1.004	(0.844,1.194)	0.968	1.005	(0.845,1.196)	0.955	1.008	(0.847,1.198)	0.931
0-5	1.016	(0.886,1.164)	0.823	1.037	(0.904,1.190)	0.606	1.013	(0.883,1.162)	0.856
15-29	1.011	(0.914,1.119)	0.827	1.016	(0.918,1.125)	0.759	1.007	(0.910,1.114)	0.899
30-54	**0.964**	**(0.915,1.014)**	**0.158**	**0.965**	**(0.916,1.016)**	**0.174**	**0.962**	**(0.914,1.013)**	**0.146**
55-64	**1.058**	**(1.008,1.111)**	**0.023**	**1.063**	**(1.013,1.116)**	**0.014**	**1.055**	**(1.004,1.107)**	**0.033**
65-74	1.053	(1.013,1.095)	0.010	**1.053**	**(1.013,1.095)**	**0.010**	**1.048**	(1.008,1.090)	0.018
≥75	**1.058**	**(1.026,1.092)**	**0.000**	**1.056**	**(1.024,1.089)**	**0.001**	**1.064**	**(1.032,1.098)**	**0.000**

Shenzhen	Air pollution,df=3			Air pollution,df=4			Air pollution,df=5		
	RR	95% CI	p	RR	95% CI	p	RR	95% CI	p
All-cause	**1.038**	**(1.003,1.074)**	**0.033**	**1.04**	**(1.014,1.067)**	**0.003**	**1.038**	**(1.004,1.074)**	**0.031**
CVD	**1.072**	**(1.015,1.131)**	**0.012**	**1.075**	**(1.018,1.134)**	**0.009**	**1.071**	**(1.014,1.13)**	**0.014**
Respiratory	**0.951**	**(0.834,1.086)**	**0.461**	**0.955**	**(0.837,1.089)**	**0.490**	**0.95**	**(0.832,1.085)**	**0.448**
Digestive	1.091	(0.897,1.328)	0.384	1.088	(0.896,1.321)	0.396	1.098	(0.901,1.337)	0.354
Endocrine and metabolic	**1.183**	**(0.919,1.523)**	**0.193**	**1.319**	**(1.006,1.73)**	**0.046**	**1.192**	**(0.927,1.534)**	**0.172**
Diabetes	**1.393**	**(0.579,3.352)**	**0.563**	**1.272**	**(0.695,2.328)**	**0.571**	**1.31**	**(0.542,3.169)**	**0.569**
Male	**1.041**	**(1.009,1.075)**	**0.013**	**1.044**	**(1.011,1.078)**	**0.009**	**1.039**	**(1.006,1.072)**	**0.020**
Female	**1.047**	**(1.003,1.093)**	**0.035**	**1.046**	**(1.002,1.092)**	**0.039**	**1.05**	**(1.006,1.096)**	**0.025**
Age (years)									
0-14	1.015	(0.865,1.19)	0.858	1.015	(0.866,1.191)	0.851	1.017	(0.868,1.192)	0.833
0-5	1.066	(0.848,1.339)	0.586	1.071	(0.852,1.347)	0.556	1.065	(0.847,1.338)	0.593
15-29	1.038	(0.967,1.115)	0.297	1.05	(0.978,1.127)	0.176	1.042	(0.971,1.119)	0.250
30-54	**1.057**	**(1.002,1.116)**	**0.044**	**1.072**	**(1.015,1.131)**	**0.013**	**1.06**	**(1.004,1.119)**	**0.036**
55-64	**1.031**	**(0.946,1.124)**	**0.488**	**1.035**	**(0.949,1.129)**	**0.434**	**1.034**	**(0.948,1.127)**	**0.455**
65-74	1.011	(0.932,1.096)	0.795	**1.008**	**(0.93,1.093)**	**0.848**	**1.011**	(0.932,1.097)	0.789
≥75	**1.076**	**(1.005,1.152)**	**0.036**	**1.074**	**(1.003,1.149)**	**0.041**	**1.072**	**(1.002,1.148)**	**0.045**

Chongqing	air pollution， df=3			date			air pollution， df=5		
	RR	95% CI	p	RR	95% CI	p	RR	95% CI	p
All-cause	**1.051**	**(1.011,1.093)**	**0.016**	**1.055**	**(1.015,1.097)**	**0.010**	**1.06**	**(1.02,1.102)**	**0.005**
CVD	**1.074**	**(1.006,1.146)**	**0.041**	**1.069**	**(1.002,1.141)**	**0.050**	**1.06**	**(0.993,1.131)**	**0.099**
Respiratory	**1.014**	**(0.918,1.121)**	**0.780**	**1.009**	**(0.914,1.115)**	**0.856**	**1.007**	**(0.911,1.112)**	**0.899**
Digestive	1.202	(1.001,1.443)	0.066	1.236	(1.029,1.485)	0.035	1.231	(1.025,1.480)	0.038
Endocrine and metabolic	**1.237**	**(1.014,1.510)**	**0.052**	**1.236**	**(1.012,1.509)**	**0.050**	**1.216**	**(0.997,1.482)**	**0.077**
Diabetes	**1.351**	**(1.085,1.683)**	**0.011**	**1.292**	**(1.039,1.606)**	**0.026**	**1.326**	**(1.069,1.645)**	**0.014**
Male	**1.058**	**(1.009,1.109)**	**0.026**	**1.053**	**(1.004,1.104)**	**0.041**	**1.067**	**(1.017,1.118)**	**0.012**
Female	**1.061**	**(1.011,1.114)**	**0.023**	**1.062**	**(1.012,1.114)**	**0.021**	**1.056**	**(1.006,1.109)**	**0.034**
Age (years)									
0-14	0.932	(0.674,1.289)	0.679	0.934	(0.676,1.291)	0.687	0.936	(0.678,1.293)	0.698
0-5	0.925	(0.448,1.909)	0.852	0.964	(0.467,1.988)	0.927	0.918	(0.444,1.899)	0.856
15-29	1.015	(0.733,1.406)	0.929	1.014	(0.732,1.406)	0.933	1	(0.721,1.388)	0.998
30-54	**1.159**	**(1.054,1.275)**	**0.004**	**1.149**	**(1.045,1.263)**	**0.006**	**1.148**	**(1.045,1.263)**	**0.006**
55-64	**1.104**	**(0.995,1.225)**	**0.070**	**1.112**	**(1.003,1.233)**	**0.050**	**1.127**	**(1.015,1.251)**	**0.036**
65-74	1.098	(1.019,1.182)	0.019	**1.083**	**(1.004,1.168)**	**0.046**	**1.115**	(1.035,1.202)	0.007
≥75	**1.05**	**(1.002,1.100)**	**0.047**	**1.057**	**(1.009,1.107)**	**0.024**	**1.054**	**(1.006,1.104)**	**0.031**

Note: Male, female and age group specific results presented for all-cause mortality.
